# Analysis of risk factors for hemorrhagic transformation after mechanical thrombectomy in acute anterior circulation large vessel occlusion stroke and construction of a nomogram prediction model

**DOI:** 10.3389/fneur.2026.1852587

**Published:** 2026-05-29

**Authors:** Mujie Yao, Keqi Lei, Yue Wan

**Affiliations:** 1 School of Medicine, Wuhan University of Science and Technology, Wuhan, China; 2 Department of Neurology, Hubei No. 3 People’s Hospital of Jianghan University, Wuhan, China

**Keywords:** acute ischemic stroke, glucose-to-lymphocyte ratio, hemorrhagic transformation, mean platelet volume, mechanical thrombectomy

## Abstract

**Objective:**

To identify risk factors for hemorrhagic transformation (HT) after mechanical thrombectomy (MT) in patients with acute anterior circulation large vessel occlusion (LVO) and to develop a predictive nomogram.

**Methods:**

This retrospective study enrolled 193 patients with acute anterior circulation LVO who underwent MT at a single center between January 2023 and December 2025. Patients were categorized into HT (*n* = 45, 23.32%) and non-HT (*n* = 148, 76.68%) groups based on postoperative imaging at 24–72 h. Univariate analysis was performed to compare baseline characteristics, biochemical indicators, and clinical variables between the two groups. Variables with *p* < 0.05 were subjected to Elastic Net regression with 10-fold cross-validation (StratifiedKFold) for variable selection and dimensionality reduction, with the optimal hyperparameters determined as C = 1 and l1_ratio = 0.9 (cross-validation AUC = 0.8615). Eight variables were retained and subsequently entered into binary logistic regression with forward stepwise selection to identify independent risk factors and construct a prediction model. A nomogram was developed and evaluated using receiver operating characteristic (ROC) curve, calibration curve with bootstrap validation (1,000 resamples), and decision curve analysis (DCA).

**Results:**

Six independent risk factors for HT were identified: history of alcohol consumption (OR = 6.423, 95% CI 2.224–18.552, *p* = 0.001), history of leukoencephalopathy (OR = 4.555, 95% CI 1.664–12.469, *p* = 0.003), elevated blood glucose-to-lymphocyte ratio (GLR) (OR = 1.105, 95% CI 1.034–1.181, *p* = 0.003), elevated D-dimer (OR = 1.102, 95% CI 1.029–1.180, *p* = 0.005), elevated venous blood glucose (OR = 1.201, 95% CI 1.037–1.390, *p* = 0.014), and reduced mean platelet volume (MPV) (OR = 0.704, 95% CI 0.537–0.925, *p* = 0.012). The nomogram demonstrated favorable discriminative ability with an AUC of 0.880 (95% CI 0.820–0.939), sensitivity of 0.933, and specificity of 0.696. Calibration curve analysis indicated good model fit (χ^2^ = 9.059, *p* = 0.337). Decision curve analysis revealed a net benefit rate > 0 when the threshold probability ranged from 0.01 to 0.97.

**Conclusion:**

History of alcohol consumption, leukoencephalopathy, elevated GLR, D-dimer, and venous blood glucose, and reduced MPV are independent risk factors for HT after MT in acute anterior circulation LVO stroke. The constructed nomogram exhibits good discrimination, calibration, and clinical utility, providing a reliable tool for individualized risk prediction.

## 1 Introduction

Acute ischemic stroke (AIS) is a leading cause of disability and mortality worldwide. Among AIS cases, large vessel occlusion (LVO) accounts for 24–46% (1, 2); owing to its severity and the suboptimal efficacy of intravenous thrombolysis, LVO-related stroke confers approximately twice the risk of death or functional dependency compared with non-LVO stroke (3). Endovascular therapy, with mechanical thrombectomy (MT) as its cornerstone, has achieved groundbreaking advances. Multiple randomized controlled trials and meta-analyses have consistently demonstrated that adjunctive MT, combined with standard medical therapy, significantly improves functional outcomes in patients with anterior circulation large-vessel occlusion (3).

Mechanical thrombectomy has become the standard treatment for acute anterior circulation large vessel occlusion ischemic stroke; however, complications such as hemorrhagic transformation (HT) following the procedure significantly impair patients’ neurological functional prognosis and clinical outcomes (4–7). According to studies, 10 to 49.5% of patients with AIS experience hemorrhagic change following interventional thrombectomy (8). Notably, hemorrhagic transformation, both symptomatic and asymptomatic, has been shown to be a separate risk factor for poor patient outcomes (9, 10). The pathophysiological mechanisms underlying hemorrhagic transformation (HT) are multifactorial, encompassing ischemia–reperfusion injury, blood–brain barrier (BBB) disruption, inflammatory activation, and coagulation-fibrinolysis imbalance. Systemic inflammation plays a pivotal role in mediating post-ischemic microvascular injury and increasing BBB permeability. Novel composite inflammatory markers may offer a more comprehensive reflection of the complex interplay among the neuro-immune-endocrine axes. Meanwhile, readily available pre-procedural laboratory parameters—such as MPV and D-dimer—are not only easily accessible but also directly involved in vascular repair and injury processes following thrombosis. However, the combined predictive value of these hematological and inflammatory indices for HT following mechanical thrombectomy remains to be fully elucidated.

To reduce the risk of HT and ultimately enhance functional recovery and long-term prognosis in stroke patients, a comprehensive investigation into the risk factors for HT following interventional thrombectomy is of significant clinical importance. Accordingly, this study retrospectively collected complete clinical and imaging data from 193 patients with acute anterior circulation large vessel occlusion who underwent interventional thrombectomy at our institution. Through univariate and multivariate regression analyses, we aimed to systematically identify and characterize independent risk factors associated with post-procedural HT.

## 2 Subjects and methods

This retrospective study was approved by the Institutional Review Board (IRB) of Hubei Provincial Third People’s Hospital (Approval No. HBZS 2026-C013-01). The requirement for informed consent was waived by the IRB as the study involved only the analysis of existing, anonymized clinical data without any additional intervention or risk to the patients. Research Subjects.

A total of 193 patients with acute anterior circulation large vessel occlusion stroke who underwent mechanical thrombectomy at Hubei Provincial Third People’s Hospital from January 2023 to December 2025 were enrolled in this study.

Inclusion criteria: (1) met the diagnostic criteria for AIS with indications for mechanical thrombectomy (11); (2) age >18 years; (3) radiologically confirmed anterior circulation large vessel occlusion suitable for thrombectomy; (4) complete clinical data. Exclusion criteria: (1) preoperative intracranial hemorrhage; (2) comorbid malignancies or severe organ failure; (3) severe organic diseases of major organs such as heart, liver, and kidney, and systemic severe infectious diseases; (4) history of bleeding (gastrointestinal or urinary tract hemorrhage) within the past month; (5) severe infections; (6) intracranial aneurysms or arteriovenous malformations; (7) brain tumors exhibiting mass effect on imaging.

### 2.1 Grouping criteria and group allocation

According to the Chinese Consensus on the Diagnosis and Treatment of Hemorrhagic Transformation after Acute Ischemic Stroke (12), HT is defined as the emergence of intracranial hemorrhage within the infarct territory or at remote sites on follow-up imaging performed 24–72 h after mechanical thrombectomy, in the absence of hemorrhage on pre-procedural CT or MRI (13). Early hyperdense lesions seen on immediate post-treatment non-contrast head CT require differentiation between HT and contrast extravasation due to their similar initial imaging appearance. This distinction is based on dynamic CT surveillance: an initial scan is routinely obtained 24 h after the procedure; if new hyperdense lesions are present, a second scan is performed at 48–72 h to evaluate their evolution. Persistent hyperdensity is diagnostic of HT, whereas marked attenuation or complete resolution indicates contrast extravasation. All patients underwent serial cranial CT within 24–72 h post-procedure. Patients with parenchymal, intraventricular, or subarachnoid hemorrhage (14) were classified into the HT group, while those without radiographic evidence of hemorrhage comprised the non-HT group.

All patients underwent interventional thrombectomy under local anesthesia in the supine position. After standard skin preparation and draping, femoral artery access was established using the Seldinger technique with an 8F arterial sheath. Complete cerebral angiography was performed to localize the occlusive lesion. A 6F guiding catheter was advanced to the vicinity of the occlusion, followed by placement of an intermediate catheter into the target vessel. A microwire was then used to navigate a microcatheter and stent retriever to the thrombus site. Once the stent was deployed, contrast injection confirmed proper positioning and full expansion. After a 5-min waiting period, the stent and catheter were withdrawn under continuous aspiration. Repeat angiography was performed to evaluate recanalization, with additional passes undertaken as needed. In cases of re-occlusion or residual thrombus, tirofiban was administered via intracatheter injection. Final angiography, performed approximately 30 min later, confirmed vessel patency, and the procedure was concluded.

Ultimately, 193 patients with AIS were enrolled in the study, of whom 45 developed hemorrhagic transformation (HT group) and 148 did not (non-HT group). Notably, this single-center study had a limited sample size (45 HT patients, 23 with sICH); thus, HT was treated as a binary imaging-based outcome without distinguishing symptomatic from asymptomatic cases.

### 2.2 Data collection

Patient data were retrospectively extracted from the electronic medical record system, including:

1. Baseline characteristics: age and sex; Alcohol consumption history, smoking history, and history of diabetes, hypertension, atrial fibrillation, leukoencephalopathy and cerebral infarction;
2. Biochemical indicators: preoperative absolute neutrophil count, preoperative absolute lymphocyte count, mean platelet volume (MPV), platelet distribution width (PDW), platelet count, D-dimer, activated partial thromboplastin time (APTT), fibrinogen, Venous blood glucose, serum potassium, uric acid (UA); neutrophil-to-lymphocyte ratio (NLR), triglyceride-glucose index (TyG), atherogenic index of plasma (AIP), blood glucose-to-lymphocyte ratio (GLR), and systemic immune-inflammation index (SII);
3. Other relevant indicators: National Institute of Health Stroke Scale (NIHSS) score, occlusion site, preoperative intravenous thrombolysis, preoperative antiplatelet drug use, preoperative anticoagulant use, preoperative pulmonary infection, arterial thrombolysis.

### 2.3 Statistical methods

#### 2.3.1 Univariate analysis

Data analysis was performed with SPSS version 27.0. Categorical data were expressed as number of cases (%) and compared between groups using the chi-square test. Normally distributed continuous variables were presented as mean ± standard deviation (x̄ ± s) and compared with the independent-samples t-test. Continuous variables that did not follow a normal distribution were expressed as median M (P25, P75) and compared using the Mann–Whitney U test. Variables with *p* < 0.05 in the univariate analysis were entered into the subsequent multivariate analysis.

#### 2.3.2 Elastic net regression with 10-fold cross-validation

Variables with *p* < 0.05 from the univariate analysis were incorporated into an Elastic Net regression for dimensionality reduction. Ten-fold cross-validation (utilizing StratifiedKFold sampling) was employed to determine the optimal regularization parameters, and variables with non-zero coefficients were retained, using the ROC-AUC as the evaluation metric.

#### 2.3.3 Binary logistic regression

For the candidate variables, binary logistic regression analysis was performed using the forward stepwise selection method (inclusion criterion: *p* < 0.05) to construct a bleeding risk prediction model, and the odds ratio (OR) and 95% confidence interval (CI) for each variable were calculated.

#### 2.3.4 Nomogram construction and model evaluation

Based on the statistical results, a nomogram model for predicting the risk of hemorrhagic transformation (HT) after mechanical thrombectomy was constructed using the rms package in R software (version 4.5.1). The discriminative ability of the nomogram was evaluated by plotting a receiver operating characteristic (ROC) curve. Goodness-of-fit was assessed with the bootstrap method (1,000 resamples), and a calibration curve was drawn using R software (version 4.5.1). The clinical applicability of the nomogram was examined with a decision curve plotted via the rmda package in R software (version 4.5.1). A two-sided test with *α* = 0.05 was applied throughout.

## 3 Results

### 3.1 Univariate analysis of factors influencing hemorrhagic transformation after thrombectomy in AIS patients

This study included 193 patients: 148 in the non-hemorrhagic transformation group (76.68%) and 45 in the hemorrhagic transformation group (23.32%). Comparisons between the two groups showed no statistically significant differences in gender distribution, smoking history, diabetes history, atrial fibrillation history, cerebral infarction history, intravenous thrombolysis status, arterial thrombolysis, antiplatelet drug use, anticoagulant drug use, occlusion site, TyG index, AIP platelet count, admission NIHSS score, LDL-C level, age, absolute neutrophil count, PDW, UA level, D-dimer level, APTT, fibrinogen level, C-reactive protein level, or serum potassium level (*p* > 0.05). Statistically significant differences existed in the distributions of absolute lymphocyte count, MPV, NLR, GLR, SII, D-dimer, blood glucose, Alcohol consumption history, hypertension history, preoperative pulmonary infection, and leukoencephalopathy history between the two groups (*p* < 0.05), as detailed in Tables 1, 2.

**Table 1:** Comparison of categorical data between the HT and non-HT groups.

Variable	HT group (*n* = 45)	Non-HT group (*n* = 148)	χ2	*p*
Sex (Male/Female)	15/30	57/91	0.396	0.529
Smoking history [*n* (%)]	20 (44.44)	48 (32.43)	2.182	0.14
Alcohol consumption history [*n* (%)]	18 (40.00)	23 (15.54)	12.34	<0.001
History of hypertension [*n* (%)]	23 (51.11)	101 (68.24)	4.409	0.036
History of diabetes [*n* (%)]	11 (24.44)	30 (20.27)	0.359	0.549
Preoperative pulmonary infection [*n* (%)]	20 (44.44)	32 (21.62)	9.132	0.003
History of atrial fibrillation [*n* (%)]	19 (42.22)	45 (30.41)	2.174	0.14
Leukoencephalopathy [*n* (%)]	19 (42.22)	20 (13.51)	17.639	0
History of cerebral infarction [*n* (%)]	7 (15.56)	26 (17.57)	0.099	0.754
Intravenous thrombolysis [*n* (%)]	11 (24.44)	50 (33.78)	1.392	0.238
Intra-arterial thrombolysis [*n* (%)]	35 (77.78)	130 (87.84)	2.816	0.093
Antiplatelet medication use [*n* (%)]	10 (22.22)	19 (12.84)	2.38	0.123
Anticoagulant medication use [*n* (%)]	9 (20.00)	24 (16.22)	0.349	0.555
Occlusion site [*n* (%)]				
Middle cerebral artery	26 (57.78)	102 (68.92)	6.215	0.184
Middle cerebral artery, anterior cerebral artery	1 (2.22)	2 (1.35)		
Anterior cerebral artery	0 (0.00)	2 (1.35)		
Internal carotid artery	17 (37.78)	32 (21.62)		
Internal carotid artery, middle cerebral artery	1 (2.22)	10 (6.76)		

**Table 2:** Comparison of measurement data between the HT group and the non-HT group.

Variable	HT group (*n* = 45)	Non-HT group (*n* = 148)	t/z	*p*
TyG ( $\bar{x}\pm s$ )	1.62 ± 0.72	1.43 ± 0.71	−1.516	0.131
AIP ( $\bar{x}\pm s$ )	−0.06 ± 0.27	0.03 ± 0.33	1.559	0.121
Platelet count ( $\bar{x}\pm s$ )	197.31 ± 61.88	215.34 ± 66.41	1.619	0.107
Admission NIHSS score ( $\bar{x}\pm s$ )	16.58 ± 6.16	15.12 ± 6.49	−1.333	0.184
LDL-C ( $\bar{x}\pm s$ )	2.46 ± 0.74	2.51 ± 0.99	0.377	0.707
Age [M (P25, P75), years]	72.000 (62.0, 79.5)	68.000 (61.0, 75.0)	−1.188	0.235
Neutrophils [M (P25, P75)]	7.360 (4.7, 9.3)	5.815 (4.6, 8.1)	−1.806	0.071
Lymphocytes [M (P25, P75)]	0.820 (0.5, 1.2)	1.335 (0.9, 2.0)	−3.532	<0.001
MPV[M(P25, P75), fL]	9.600 (9.1, 10.1)	10.400 (9.5, 11.8)	−3.185	0.001
PDW[M(P25, P75), fL]	16.000 (14.4, 16.5)	15.900 (10.6, 16.3)	−1.007	0.314
UA[M(P25, P75), μmol/L]	311.200 (221.9, 393.9)	306.135 (244.0, 394.0)	−0.34	0.734
NLR[M(P25, P75)]	8.385 (4.0, 15.1)	4.706 (2.6, 8.1)	−3.326	0.001
SII[M(P25, P75)]	1547.590 (889.8, 3056.2)	920.450 (581.8, 1630.7)	−2.839	0.005
GLR[M(P25, P75)]	10.760 (6.9, 18.8)	5.497 (3.4, 9.4)	−4.992	0.000
D-dimer [M (P25, P75), mg/L]	1.980 (0.8, 10.4)	1.005 (0.5, 2.9)	−2.988	0.003
APTT[M(P25, P75), s]	29.200 (25.0, 43.0)	28.400 (25.9, 33.1)	−0.965	0.335
Fibrinogen [M (P25, P75), g/L]	2.940 (2.5, 3.4)	2.825 (2.4, 3.4)	−0.594	0.552
C-reactive protein [M (P25, P75), mg/L]	10.200 (2.7, 27.2)	5.550 (1.6, 17.6)	−1.841	0.066
Venous blood glucose [M (P25, P75), mmol/L]	9.350 (7.6, 13.6)	7.065 (6.2, 8.4)	−5.016	<0.001
Serum potassium [M (P25, P75), mmol/L]	4.010 (3.6, 4.2)	3.980 (3.6, 4.3)	−0.465	0.642

### 3.2 Elastic net regression with 10-fold cross-validation

Variables with a *p*-value of <0.05 in the univariate analysis underwent variable selection and dimensionality reduction. Optimal hyperparameters were determined via 10-fold cross-validation (utilizing StratifiedKFold for stratified sampling); the best hyperparameters identified through grid search were “C = 1” and “l1_ratio = 0.9,” at which point the model achieved an AUC of 0.8615 during cross-validation. Ultimately, eight variables were selected and retained in the model, ranked by importance as follows: leukoencephalopathy history (0.181), Alcohol consumption history (0.160), blood glucose (0.146), GLR (0.137), D-dimer (0.125), mean platelet volume (−0.117), absolute lymphocyte count (−0.058), and NLR (0.032).

### 3.3 Binary logistic regression analysis of factors influencing hemorrhagic transformation following thrombectomy in AIS patients

Binary logistic regression analysis was performed with post-thrombectomy hemorrhagic transformation occurrence in AIS patients as the dependent variable (assigned: present = 1, absent = 0), and Alcohol consumption history (assigned: yes = 1, no = 0), leukoencephalopathy history (assigned: yes = 1, no = 0), absolute lymphocyte count (actual value), MPV (actual value), NLR (actual value), GLR (actual value), D-dimer (actual value) and blood glucose (actual value) as independent variables. The results demonstrated that Alcohol consumption history, leukoencephalopathy history, elevated GLR at admission, high D-dimer levels, hyperglycemia, and low mean platelet volume were independent risk factors for HT occurrence, as detailed in Table 3.

**Table 3:** Results of binary logistic regression analysis on factors influencing hemorrhagic transformation occurrence in AIS patients after thrombectomy.

Independent variables	β	SE	Wald χ2	*p*	OR	OR(95% CI)
Alcohol consumption history	1.86	0.541	11.81	0.001	6.423	2.224 ~ 18.552
Leukoencephalopathy	1.516	0.514	8.709	0.003	4.555	1.664 ~ 12.469
MPV	−0.351	0.139	6.376	0.012	0.704	0.537 ~ 0.925
GLR	0.1	0.034	8.741	0.003	1.105	1.034 ~ 1.181
D-dimer	0.097	0.035	7.773	0.005	1.102	1.029 ~ 1.180
Venous blood glucose	0.183	0.075	6.001	0.014	1.201	1.037 ~ 1.390

### 3.4 Construction and validation of a nomogram model for predicting post-thrombectomy HT risk in AIS patients

**Figure 1:**
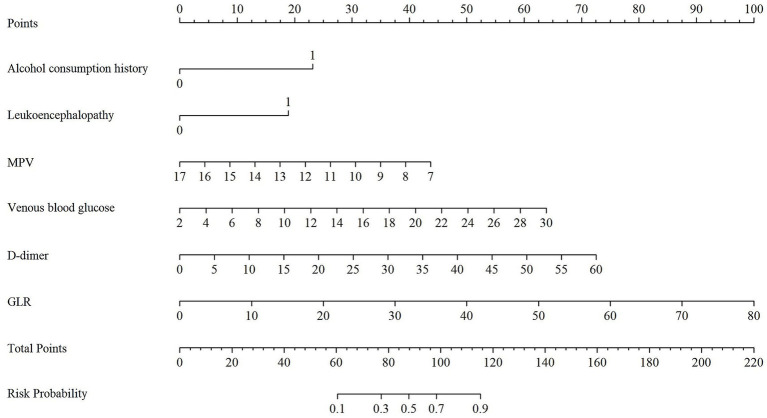
Nomogram for predicting hemorrhagic transformation risk after mechanical thrombectomy.

**Figure 2:**
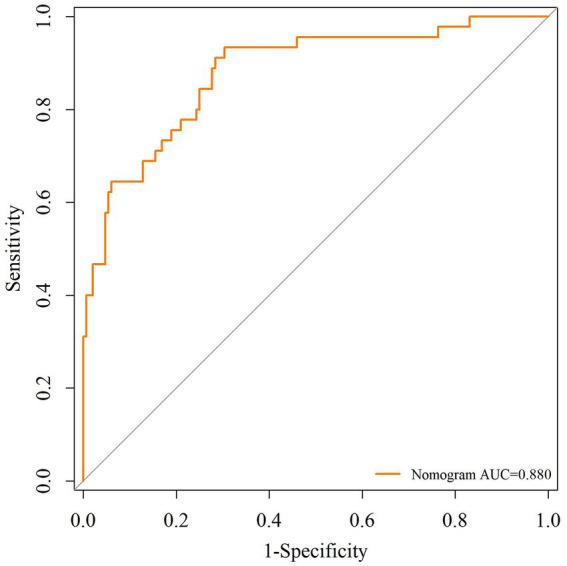
ROC curve of the nomogram.

**Figure 3:**
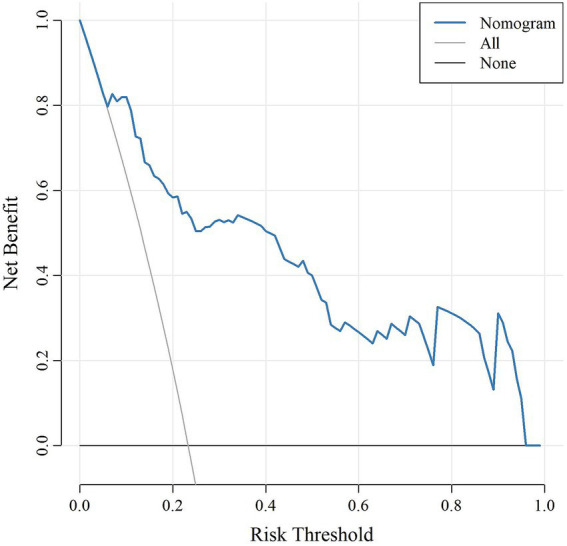
Calibration curve of the nomogram.

**Figure 4:**
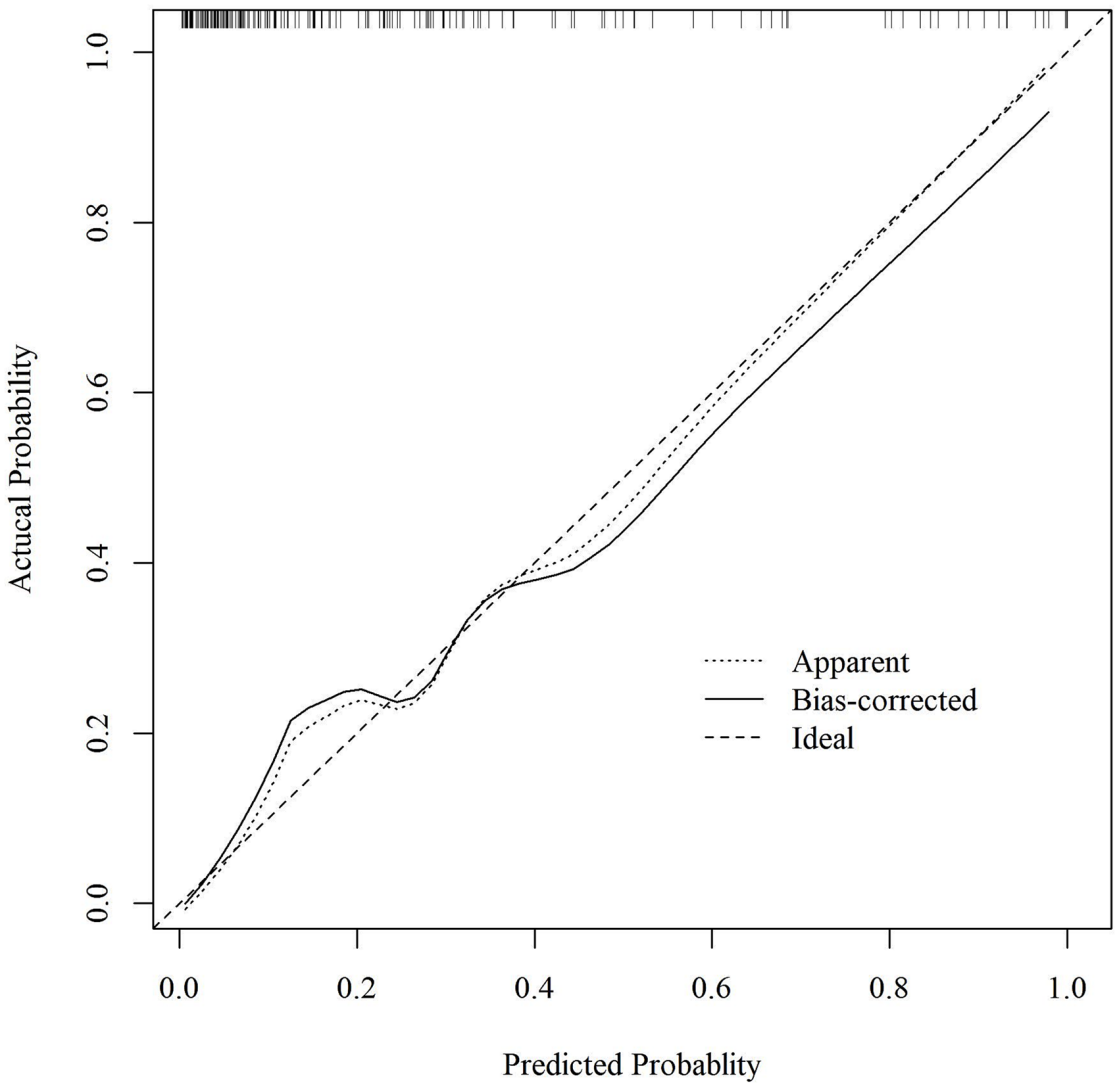
Decision curve of the nomogram.

Based on the binary logistic regression analysis results, a nomogram model was constructed to predict HT risk following thrombectomy in AIS patients, as shown in Figure 1. ROC curve analysis indicated that the nomogram model achieved an AUC of 0.880 [95% CI (0.820 ~ 0.939)] for predicting post-thrombectomy HT in AIS patients, with a sensitivity of 0.933 and specificity of 0.696, as presented in Figure 2. Calibration curve analysis demonstrated good fit for the nomogram model (χ^2^ = 9.059, *p* = 0.337), as shown in Figure 3. Decision curve analysis indicated a net benefit rate >0 for the nomogram model when the threshold probability ranged from 0.01 to 0.97, see Figure 4. For instance, consider an 88-year-old male with a history of chronic alcohol consumption and white matter lesions on imaging. His laboratory findings were as follows: blood glucose 9.79 mmol/L, mean platelet volume 10.1 fL, D-dimer 2.74 μg/mL, and GLR 13.79. Using the nomogram, each variable was assigned a score—23 points for alcohol history, 19 for white matter lesions, 8 for blood glucose, 11 for mean platelet volume, 3 for D-dimer, and 17 for GLR—yielding a total score of 81. This corresponds to an estimated bleeding probability of approximately 48–50%, placing the patient in a moderately high-risk category. Accordingly, he was classified into the bleeding group in this study.

## 4 Discussion

This single-center retrospective study identified Alcohol consumption history, leukoencephalopathy, elevated admission blood glucose-to-lymphocyte ratio, D-dimer and blood glucose levels, and lower mean platelet volume as independent factors associated with hemorrhagic transformation development after mechanical thrombectomy in acute anterior circulation large vessel occlusion stroke patients.

Leukoencephalopathy manifests radiologically as leukoaraiosis (LA), typically associated with cerebral small vessel disease. This structural abnormality may increase vascular wall fragility, elevating peri-procedural and postprocedural hemorrhage risk (15, 16). The development of LA is associated with disruption of blood–brain barrier permeability (17), which also constitutes a key mechanism in hemorrhagic transformation (18). These findings are consistent with previous research.

The interaction between inflammation and thrombosis constitutes an important pathological basis for the occurrence and development of hemorrhagic transformation (19). When cerebral embolism occurs, local tissue ischemia and hypoxia rapidly activate the inflammatory response. Numerous pro-inflammatory cytokines are released (20), attracting inflammatory cells such as neutrophils and monocytes to aggregate (16), and compromising the integrity of the blood–brain barrier (21).

The Glucose to Lymphocyte Ratio (GLR) is a novel marker of inflammatory burden (22), reflecting the coexistence of hyperglycemia and lymphocyte decrease. Hyperglycemia has been identified by multiple studies as an independent predictor of hemorrhagic transformation (HT) through promoting oxidative stress and activating matrix metalloproteinases (MMPs) to disrupt the blood–brain barrier (23). Lymphopenia reflects the intensity of post-stroke immunosuppression and systemic inflammatory response (24). The Glucose-to-Lymphocyte Ratio (GLR) integrates information from both dimensions. Its prognostic value in critical illnesses such as myocardial infarction and acute pancreatitis (25–28) has been validated, serving as an independent predictor of long-term all-cause mortality (25), with predictive efficacy superior to blood glucose or lymphocyte count alone (27). This study is the first to identify GLR as an independent risk factor for hemorrhagic transformation after mechanical thrombectomy in AIS patients, which requires further validation in the future.

Mean platelet volume (MPV) is used not only to evaluate platelet function and activation levels but also to differentiate between high and low degrees of vascular inflammation. Platelet levels directly affect coagulation, vascular injury, and inflammatory response. Moreover, activated platelets provide the active mediators required for inflammatory responses and coagulation (29). During acute ischemia, under stimulation by substances such as epinephrine, platelets express increased adhesion receptor glycoprotein GPIb/Ia, which accelerates enzymatic reactions and metabolism, enhancing platelet adhesion and aggregation and consequently leading to thrombus formation. An elevated mean platelet volume (MPV) typically indicates increased platelet size and enhanced activity, but its level shows a negative correlation with the severity of inflammation. This phenomenon occurs due to the chemotactic property of platelets during inflammatory responses, which causes platelets to concentrate in areas of high inflammation and gradually deplete, resulting in decreased MPV (29–31). This study identified low MPV as a relevant risk factor for hemorrhagic transformation (HT) following mechanical thrombectomy. On one hand, reduced platelet activity may impair inter-platelet adhesion and aggregation, diminishing their ability to effectively exert hemostatic effects upon vascular endothelial injury. On the other hand, decreased MPV may reflect systemic inflammation, where the recruitment, activation, and infiltration of inflammatory cells contribute to blood–brain barrier disruption (32), thereby promoting HT development.

This study indicates that Alcohol consumption history is a risk factor for hemorrhagic transformation (HT). Alcohol can affect vascular walls, platelets, and coagulation factors through multiple mechanisms, thereby inducing HT. Alcohol not only alters platelet lipid and fatty acid content, causing prostaglandin synthesis dysfunction and impaired platelet function (33, 34), but also reduces circulating platelet counts by affecting mitochondrial gene-mediated apoptosis processes (35). Excessive alcohol consumption decreases nitric oxide (NO) levels while increasing endogenous NO inhibitors such as asymmetric dimethylarginine (ADMA), leading to impaired vascular endothelial relaxation (36). It also directly affects vascular endothelial cells, triggering inflammatory responses in the vascular wall (37), ultimately resulting in vascular wall damage. Alcohol metabolism generates substantial reactive oxygen species that disrupt redox homeostasis, inducing liver injury (38). Concurrently, acetaldehyde derived from alcohol in the intestine compromises the gut barrier, enabling endotoxin translocation into systemic circulation and causing hepatic damage (39).

This impairment directly compromises coagulation factor synthesis. Studies demonstrate that heavy alcohol consumption (>4 drinks/day) markedly elevates risks of intracerebral hemorrhage and subarachnoid hemorrhage (40). Consequently, acute ischemic stroke (AIS) patients with chronic heavy Alcohol consumption history warrant intensified monitoring for hemorrhagic transformation (HT) following mechanical thrombectomy (MT). However, in the present study, alcohol consumption history was ascertained from medical records based on attending physicians’ subjective assessments. Consequently, this variable was operationalized as a binary measure (habitual drinking: yes or no), without quantification of intake volume, frequency, or duration. Our findings suggest that alcohol consumption may constitute a risk factor for hemorrhagic transformation following mechanical thrombectomy. Future studies should employ standardized criteria—such as the NHANES alcohol consumption definitions—to enable more granular characterization of drinking behaviors, including quantification of intake volume, frequency, and beverage type.

## 5 Conclusion and limitations

In summary, Alcohol consumption history, leukoencephalopathy, higher admission blood glucose-to-lymphocyte ratio, elevated D-dimer and blood glucose levels, and lower mean platelet volume constitute risk factors for hemorrhagic transformation following mechanical thrombectomy in acute anterior circulation large vessel occlusion stroke. The nomogram prediction model incorporating these factors exhibits favorable discrimination, calibration, and clinical utility for predicting post-thrombectomy hemorrhagic transformation.

By integrating multidimensional variables—including clinical history, inflammatory markers, coagulation parameters (blood glucose), and platelet indices—this model more comprehensively reflects patients’ overall risk profiles compared to those utilizing single or limited indicators, demonstrating substantial potential for clinical translation.

This was a single-center, retrospective study with a limited sample size. The number of HT events (*n* = 45) is relatively small compared to the number of candidate predictors, increasing the risk of overfitting. Although internal validation (bootstrap) was performed, the model’s stability and generalizability require external validation in larger, prospective, multi-center cohorts. The current nomogram should be considered an exploratory tool.

Furthermore, this study incorporated readily available preoperative laboratory markers, demographic data, and intraoperative angiographic findings to comprehensively assess the risk of post-procedural hemorrhagic transformation. This approach offers dual clinical benefits: first, it assists interventional physicians in evaluating the necessity of thrombectomy; second, by identifying high-risk patients, it enables clinicians to judiciously delay antiplatelet or anticoagulant resumption, thereby optimizing clinical outcomes.

Notably, early venous filling and the basal ganglia blush sign are established angiographic markers of poor prognosis following endovascular treatment, as they are associated with irreversible infarction and post-procedural hemorrhage. However, these specific indicators were not included in the present study’s scope.

This study did not capture blood pressure management details—whether pre-, intra-, or post-procedure—in patients undergoing mechanical thrombectomy, as such data were unavailable. Because data were collected retrospectively from electronic medical records and patients received only 24-h ambulatory blood pressure monitoring after the procedure, complete retrieval of perioperative blood pressure profiles was not feasible. Future studies should therefore consider analyzing blood pressure at specific postoperative time points, such as 24 and 48 h, to better elucidate its impact on outcomes.

## Data Availability

The original contributions presented in the study are included in the article/supplementary material, further inquiries can be directed to the corresponding author.
